# Fe(ii) and Fe(iii) dithiocarbamate complexes as single source precursors to nanoscale iron sulfides: a combined synthetic and *in situ* XAS approach†‡

**DOI:** 10.1039/c9na00262f

**Published:** 2019-06-05

**Authors:** Anna Roffey, Nathan Hollingsworth, Husn-Ubayda Islam, Wim Bras, Gopinathan Sankar, Nora H. de Leeuw, Graeme Hogarth

**Affiliations:** Department of Chemistry, King's College London Britannia House, 7 Trinity Street London SE1 1DB UK; Department of Chemistry, University College London 20 Gordon Street London WC1H OAJ UK; Netherlands Organisation for Scientific Research DUBBLE@ESRF 38043 Grenoble France; Chemistry Division, Oak Ridge National Laboratory Oak Ridge Tennessee 37831 USA; School of Chemistry, Cardiff University Main Building, Park Place Cardiff CF10 3AT UK

## Abstract

Nanoparticulate iron sulfides have many potential applications and are also proposed to be prebiotic catalysts for the reduction of CO_2_ to biologically important molecules, thus the development of reliable routes to specific phases with controlled sizes and morphologies is important. Here we focus on the use of iron dithiocarbamate complexes as single source precursors (SSPs) to generate greigite and pyrrhotite nanoparticles. Since these minerals contain both iron(iii) and iron(ii) centres, SSPs in both oxidation states, [Fe(S_2_CNR_2_)_3_] and *cis*-[Fe(CO)_2_(S_2_CNR_2_)_2_] respectively, have been utilised. Use of this Fe(ii) precursor is novel and it readily loses both carbonyls in a single step (as shown by TGA measurements) providing an *in situ* source of the extremely air-sensitive Fe(ii) dithiocarbamate complexes [Fe(S_2_CNR_2_)_2_]. Decomposition of [Fe(S_2_CNR_2_)_3_] alone in oleylamine affords primarily pyrrhotite, although by careful control of reaction conditions (*ca.* 230 °C, 40–50 nM SSP) a window exists in which pure greigite nanoparticles can be isolated. With *cis*-[Fe(CO)_2_(S_2_CNR_2_)_2_] we were unable to produce pure greigite, with pyrrhotite formation dominating, a similar situation being found with mixtures of Fe(ii) and Fe(iii) precursors. *In situ* X-ray absorption spectroscopy (XAS) studies showed that heating [Fe(S_2_CN^i^Bu_2_)_3_] in oleylamine resulted in amine coordination and, at *ca.* 60 °C, reduction of Fe(iii) to Fe(ii) with (proposed) elimination of thiuram disulfide (S_2_CNR_2_)_2_. We thus carried out a series of decomposition studies with added thiuram disulfide (R = ^i^Bu) and found that addition of 1–2 equivalents led to the formation of pure greigite nanoparticles between 230 and 280 °C with low SSP concentrations. Average particle size does not vary significantly with increasing concentration, thus providing a convenient route to *ca.* 40 nm greigite nanoparticles. *In situ* XAS studies have been carried out and allow a decomposition pathway for [Fe(S_2_CN^i^Bu_2_)_3_] in oleylamine to be established; reduction of Fe(iii) to Fe(ii) reduction triggers substitution of the secondary amide backbone by oleylamine (RNH_2_) resulting in the *in situ* formation of a primary dithiocarbamate derivative [Fe(RNH_2_)_2_(S_2_CNHR)_2_]. This in turn extrudes RNCS to afford molecular precursors of the observed FeS nanomaterials. The precise role of thiuram disulfide in the decomposition process is unknown, but it likely plays a part in controlling the Fe(iii)–Fe(ii) equilibrium and may also act as a source of sulfur allowing control over the Fe : S ratio in the mineral products.

## Abstract 2

Fe(ii) and Fe(iii) dithiocarbamates are precursors to a range of nanoparticulate iron sulfides and *in situ* studies give insight into the molecular decomposition mechanism being dominated by reductive-elimination of thiuram disulfide and amide-exchange.

## Graphical abstract

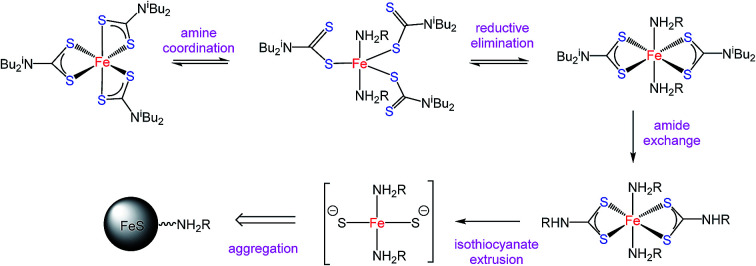

## Introduction

Several phases of iron sulfide are known including; mackinawite (FeS), troilite (FeS) greigite (Fe_3_S_4_), pyrrhotite (Fe_1−*x*_S, commonly Fe_7_S_8_ and Fe_8_S_9_), marcasite (orthorhombic FeS_2_) and pyrite (cubic FeS_2_). While some contain only Fe(ii) others, such as greigite, contain both Fe(ii) and Fe(iii).^1^ Nanoscale iron sulfides have potential applications as hydrogen evolution catalysts,^2^ semiconductor materials for solar cells,^3,4^ photodiode materials,^5^ photocatalysts and sensors,^6^ information storage,^7^ and in biomedicine.^8–10^ They are also implicated in prebiotic chemistry,^11,12^ a widely considered hypothesis being that iron sulfides in the chimney cavities of hydrothermal vents^13^ catalysed CO_2_ reduction forming a primitive acetyl-CoA pathway similar to that in contemporary enzymes.^14–16^ Greigite is structurally similar to the Fe_4_S_4_ cluster sub-units found in ferredoxins^17^ which have been shown to act as electron-transfer sites and to be catalytically active centers for molecular transformations.^18^ These enzymes are highly product-specific and efficient, as shown for example in formate dehydrogenases, which are able to reduce CO_2_ to formate under moderate conditions.^19–24^ The catalytic nature of greigite in CO_2_ activation has been demonstrated,^25,26^ while iron sulfides have also been shown to catalyse CO_2_ reduction^27^ which in the presence of H_2_S can lead to a range of thiols.^28^

In a recent perspective review,^29^ O'Brien and co-workers considered three methods for the synthesis of nanoparticulate iron sulfides; hydrothermal, solvent-free and solvothermal processes. The latter, which utilise single source precursors (SSPs), are particularly attractive as the ratio of iron to sulfur can be tuned.^30–45^ In 2008, O'Brien reported that solvothermal decomposition of [N^*n*^Bu_4_]_2_[Fe_4_S_4_(SPh)_4_] provided a convenient route to iron sulfide nanomaterials, tuning of the reaction medium and temperature leading to selective formation of different phases.^37^ Thus at 180 °C in octylamine, pyrrhotite nanoparticles result, while at 230 °C in oleylamine greigite nanoparticles are formed. That pyrrhotite is generated at low and greigite at high temperatures is particularly interesting as the cluster SSP has an Fe_4_S_4_ core that is similar to the repeating unit of greigite. Thus it is feasible that the molecular geometry of the precursor directs the nanoparticle growth at higher temperatures where decomposition is fast. In related work, Tilley and co-workers reported the synthesis of greigite nanocrystals from the hot-injection of [{Fe(*N*-MeIm)_6_}S_8_] (*N*-MeIm = *N*-methylimidazole) into oleylamine at 300 °C.^38^ Heating the same SSP for longer periods gave mixtures of greigite and pyrrhotite, while upon prolonged heating (4 h) sub-micrometer crystallites of pure pyrrhotite were formed, suggesting that pyrrhotite was the thermally stable phase.^46^ While these approaches are elegant, the SSPs used are not easy to prepare and handle and it would be advantageous to develop SSPs that can be easily synthesised from cheap, readily available, starting materials and are air and moisture stable.

Dithiocarbamate (S_2_CNR_2_) complexes potentially provide such SSPs as the ligands themselves are easily prepared from secondary amines and CS_2_ under basic conditions in water.^47^ Further, Fe(iii) complexes [Fe(S_2_CNR_2_)_3_], are air-stable crystalline solids formed in high yields upon addition of iron salts to aqueous solutions of dithiocarbamates.^47^ Iron sulfides generally contain Fe(ii), and a range of Fe(ii) dithiocarbamate complexes of the type [Fe(S_2_CNR_2_)_2_L_2_] (*e.g.* L = CO; L_2_ = 1,10-phen) are known.^48–52^ In 2008, Gao first reported the use of iron dithiocarbamate complexes as SSPs, detailing the effects of decomposition temperature and solvent on Fe(ii) [Fe(S_2_CNEt_2_)_2_(1,10-phen)] and Fe(iii) [Fe(S_2_CNEt_2_)_3_] complexes.^41^ When [Fe(S_2_CNEt_2_)_2_(1,10-phen)] was decomposed in oleylamine for 5 min at *ca.* 260–300 °C, hexagonal nanosheets of pyrrhotite (monoclinic) were produced, while at high temperatures (320 °C) troilite (hexagonal FeS) resulted. In contrast, decomposition of [Fe(S_2_CNEt_2_)_3_] in oleylamine gave a mixture of pyrrhotite and greigite at all temperatures below 300 °C, but at 320 °C pure pyrrhotite nanosheets resulted.^41^ A closely related study by Xu, Wang and co-workers investigated the effects of solvent on the decomposition of [Fe(S_2_CNEt_2_)_2_(1,10-phen)] and [Fe(S_2_CNEt_2_)_3_].^42^ Decomposition of [Fe(S_2_CNEt_2_)_3_] in oleylamine/octadecane mixtures (1 : 1) afforded greigite nanosheets, while decomposition of [Fe(S_2_CNEt_2_)_2_(1,10-phen)] under similar conditions gave pyrrhotite nanosheets.^42^ More recently, O'Brien reported on the effects of temperature, solvent and ligand substituents on the decomposition of [Fe(S_2_CNR_2_)_3_] SSPs.^53^

In developing catalysts for electrocatalytic CO_2_ reduction^25^ we sought to expand on the work described above to prepare iron sulfide nanomaterials, in particular greigite, varying both particle phase, morphology and size. A key feature of greigite is the presence of both Fe(ii) and Fe(iii) centres, being an inverse spinel A(AB)S_4_ with Fe(ii) in the tetrahedral A sites and both Fe(ii) and Fe(iii) in octahedral B sites. Thus it seemed likely that a successful solvothermal approach would be the decomposition of a mixture of Fe(ii) and Fe(iii) SSPs. Like others we have also used Fe(iii) complexes, [Fe(S_2_CNR_2_)_3_], as Fe(iii) SSPs but utilise readily prepared dicarbonyl complexes *cis*-[Fe(CO)_2_(S_2_CNR_2_)_2_] as Fe(ii) SSPs, as they readily lose both carbonyls upon heating. We also find that heating [Fe(S_2_CNR_2_)_3_] in amines results in intramolecular electron transfer resulting in generation of otherwise difficult to access Fe(ii) bis(dithiocarbamate) complexes, [Fe(S_2_CNR_2_)_2_], and the oxidised form of dithiocarbamate namely thiuram disulfide, We can then exploit this by addition of added thiuram disulfide to the decomposing mixture leading to significant differences in products distributions from the same SSP precursors.

## Results and discussion

### (i) Synthesis and characterisation of [Fe(S_2_CNR_2_)_3_] and *cis*-[Fe(CO)_2_(S_2_CNR_2_)_2_]

**Scheme 1:**
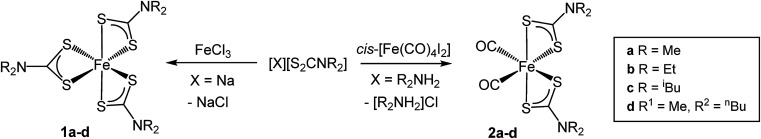
Synthesis of [Fe(S_2_CNR_2_)_3_] (1a–d) and *cis*-[Fe(CO)_2_(S_2_CNR_2_)_2_] (2a–d).

The choice of Fe(iii) SSPs was straightforward since [Fe(S_2_CNR_2_)_3_] are easily prepared according to well-established literature methods.^54^ Addition of *ca.* 3 equivalents of Na(S_2_CNR_2_) to an aqueous solution of FeCl_3_ giving [Fe(S_2_CNR_2_)_3_] (1a–d) as black-brown solids after work up (Scheme 1).

For an Fe(ii) SSP we initially considered bis(dithiocarbamate) complexes, [Fe(S_2_CNR_2_)_2_], first prepared in 1950 (ref. 55) as chocolate-brown solids,^56,57^ while in 1975 Ileperuma and Feltham reported the crystal structure of [Fe(S_2_CNEt_2_)_2_]^58^ the metal centre being square planar. They are, however, extremely air sensitive, being rapidly oxidised to [Fe(S_2_CNR_2_)_3_]. The Fe(ii) centre can be stabilised by addition of bidentate donor ligands such as 1,10-phenanthroline (phen), 2,2-bipyridine (bipy)^48,52,59^ and 1,2-bis(diphenylphosphino)ethane (dppe).^51^ While these complexes may be able to act as Fe(ii) SSPs, their high molecular weights and the non-volatile nature make them less desirable. In contrast, carbonyl derivatives, *cis*-[Fe(CO)_2_(S_2_CNR_2_)_2_] (2), are relatively air stable and can be prepared *via* a number of methods.^52,60,61^ We used a route developed by Dean^61^ involving reaction of *cis*-[Fe(CO)_4_I_2_] with two equivalents of dithiocarbamate salt. Initially the sodium salts were used but their poor solubility in organic solvents led to long reaction times and thus ammonium dithiocarbamate salts, [R_2_NH_2_][S_2_CNR_2_], were adopted instead. These were prepared upon reaction of CS_2_ with two equivalents of the chosen secondary amine and were fully characterised (see ESI‡). Addition of *ca.* 2 equivalents of [R_2_NH_2_][S_2_CNR_2_] to *cis*-[Fe(CO)_4_I_2_] in Et_2_O/CH_2_Cl_2_ at room temperature slowly (*ca.* 18 h) afforded *cis*-[Fe(CO)_2_(S_2_CNR_2_)_2_] (2a–d) in moderate (30–45%) yields after work-up. For 2c an excess of dithiocarbamate salt was used and this increased the rate of reaction dramatically. IR spectroscopy confirmed the presence of two carbonyls in a *cis* conformation, all exhibiting two peaks at *ca.* 2025 and 1965 cm^−1^ being close to values previously reported.^48^ By fast atom bombardment (FAB) mass spectrometry, only in the case of 2a was the intact molecular ion observed; in all other cases the heaviest ion was [Fe(S_2_CNR_2_)_2_], suggesting that loss of both carbonyls is a facile process, supporting our hypothesis that such complexes would be good Fe(ii) precursors.

### (ii) Thermogravimetric analysis (TGA)

Although not directly comparable to the solvothermal decomposition work (carried out in a coordinating solvent/capping agent which plays a significant role in the decomposition process-mechanism) TGA nevertheless provides useful information on the likely appropriateness of an SSP.

**Fig. 1:**
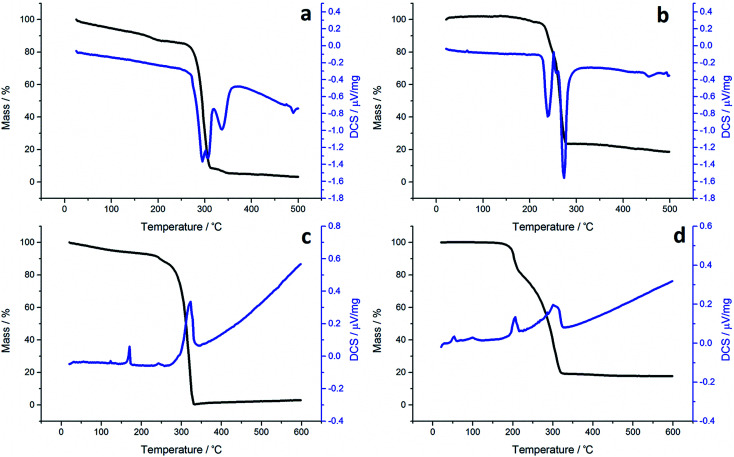
TGA (black) and DSC (blue) graphs for (a) 1a, (b) 1b, (c) 1c and (d) 1d.

**Fig. 2:**
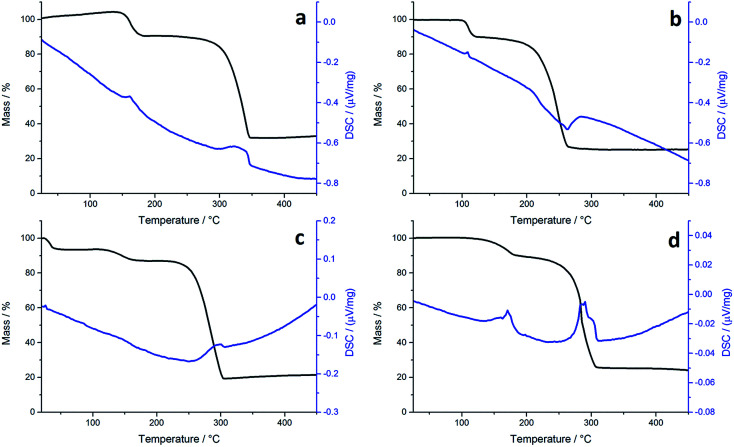
TGA (black) and DSC (blue) graphs for (a) 2a, (b) 2b, (c) 2c and (d) 2d.

TGA graphs (Fig. 1) for 1a and 1c are similar, showing thermal stability below *ca.* 300 °C, whereupon there is a sharp mass loss, a residual mass of *ca.* 3–5% remains, indicating probable sublimation. DSC for 1c shows a small peak at 170 °C indicative of melting, but no such peak is seen for 1a. Both graphs are complicated by several overlapping peaks, indicating a multistep process. TGA data for 1b and 1d differ are superficially similar, both leaving a residual mass approximately of *ca.* 20%, being close to that expected for FeS. They are some significant differences between the two. 1b initially decomposes between 226–277 °C losing 73% of its mass, followed by the gradual loss of a further 5% up to 500 °C which corresponds to the loss of a further sulfur. This differs slightly from previous work by O'Brien *et al.*, who observed a single mass loss of 79% between *ca.* 220–300 °C when they performed TGA on 1b, with no further losses up to 500 °C.^53^ The methyl-butyl derivative, 1d, decomposes between *ca.* 175–320 °C with *ca.* 81% of the mass lost. This a wider range of decomposition and a markedly lower starting temperature indicating instability due to the unsymmetrical ligand. At first glance, TGA graphs for 2a–d (Fig. 2) look similar, each showing a small mass loss at *ca.* 150 °C, followed by a much greater mass loss at *ca.* 250 °C. Both processes are sharp but occur at a temperature that is dependent on the substituents. Complex 2c appears to exhibit an additional mass loss between 25–37 °C, but this can be accounted for by the presence of co-crystallised *n*-pentane (bp 36 °C).

In all TGA graphs the initial mass decrease at *ca.* 150 °C is attributed to the loss of both carbonyls. This is followed by a period of thermal stability for the putative [Fe(S_2_CNR_2_)_2_] before it decomposes in a sharp curve. This indicates that all complexes exhibit a level of stability required for solvothermal synthesis *i.e.* they are stable at room temperature and can be delivered to the decomposition chamber, but the carbonyl ligands are labile and dissociate in a single step at a temperature lower than that at which [Fe(S_2_CNR_2_)_2_] decomposes. Consistent with mass spectral data, the most thermally stable dicarbonyl is 2a, which only begins to lose its carbonyls at *ca.* 144 °C. Likewise [Fe(S_2_CNMe_2_)_2_] is also the most thermally stable of the simple Fe(ii) complexes, decomposing at *ca.* 266 °C, some 44 °C higher than the next most stable fragment, [Fe(S_2_CN^i^Bu_2_)_2_]. Decomposition of [Fe(S_2_CNMe_2_)_2_] begins at a similar temperature to the Fe(iii) analogue 1a, indicating similar stability. However, while virtually nothing was left of 1a after decomposition, 2a decomposes to a mass approximately equal to FeS_2_. The same trend is seen between the isobutyl derivatives 2c and 1c. TGA graphs for 2b and 2d are similar, both decomposing in several steps leaving a residual mass approximately equal to FeS, the same product to which their respective Fe(iii) analogues also decompose. However, 2b, has the lowest decomposition temperature of the four Fe(ii) complexes at 180 °C, while the methyl-butyl derivative (1d) has the lowest decomposition temperature of the Fe(iii) dithiocarbamate species. The methyl-butyl Fe(ii) derivative, 2d, decomposes at a similar temperature to 2c, 42 °C higher than 1d, indicating an increased thermal stability. It should be noted that while the decompositions of the Fe(ii) bis(dithiocarbamate) complexes appear as a sharp drops in percentage mass on the TGA graph, the DSC graphs show that in all cases complexes do not fall apart in one step. The latter are complicated by overlapping peaks, some of which are endothermic (as expected when a compound decomposes) and some overall exothermic. This indicates that though decomposition is rapid, it involves several steps.

**Fig. 3:**
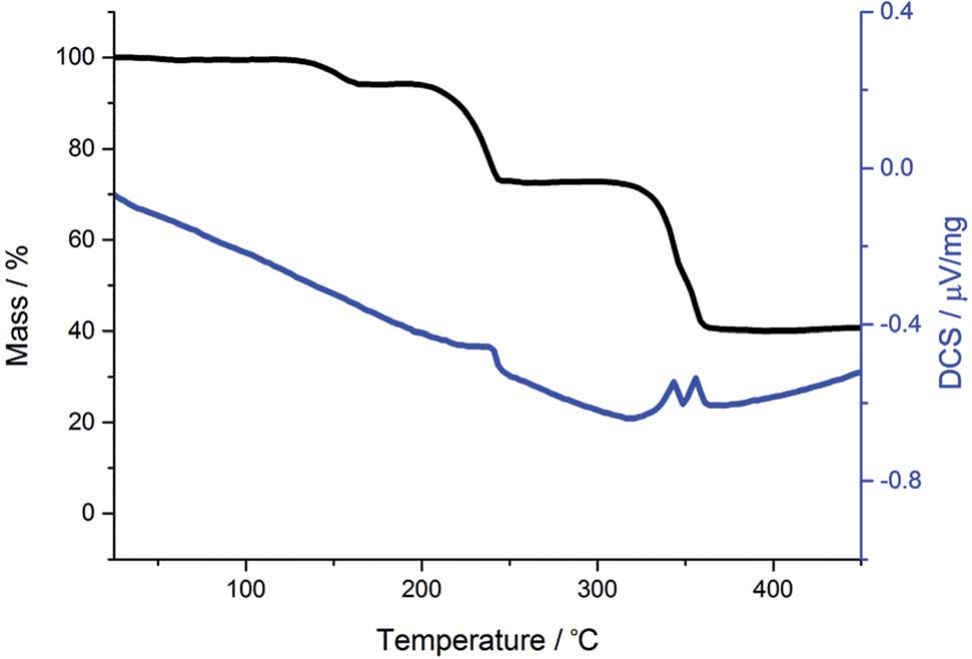
TGA (black) and DSC (blue) graphs for *cis*-[Ru(S_2_CNMe_2_)_2_(CO)_2_].

For comparison we have probed the decomposition of *cis*-[Ru(S_2_CNMe_2_)_2_(CO)_2_]^62^ (Fig. 3). Previous work has shown that *cis*-[Ru(S_2_CNR_2_)_2_(CO)_2_] decompose in solution to afford clusters containing ligands resulting from one and two carbon–sulfur bond scission processes (formally oxidative-additions).^62,63^ Unlike the iron dicarbonyl complexes, decomposition occurs in a series (four) of well-defined stages. The first mass loss at 127 °C is ascribed to loss of a single carbonyl, and this is followed by a much bigger loss at 200 °C showing now that ligand breakdown occurs before loss of the second carbonyl. The third and fourth stages occur in quick succession and are difficult to assign leaving a residual mass equivalent to RuS_2_ which is stable to 500 °C. Thus the key difference between iron and ruthenium dicarbonyl complexes is the facile loss of both carbonyls from iron, while for ruthenium a single carbonyl is lost. This behaviour is found in solution for ruthenium, heating *cis*-[Ru(CO)_2_(S_2_CNR_2_)_2_] leads to formation of dimeric [Ru(CO)(S_2_CNR_2_)(μ-S_2_CNR_2_)]_2_.^64^

### (iii) Decomposition of [Fe(S_2_CNR_2_)_3_]

**Fig. 4:**
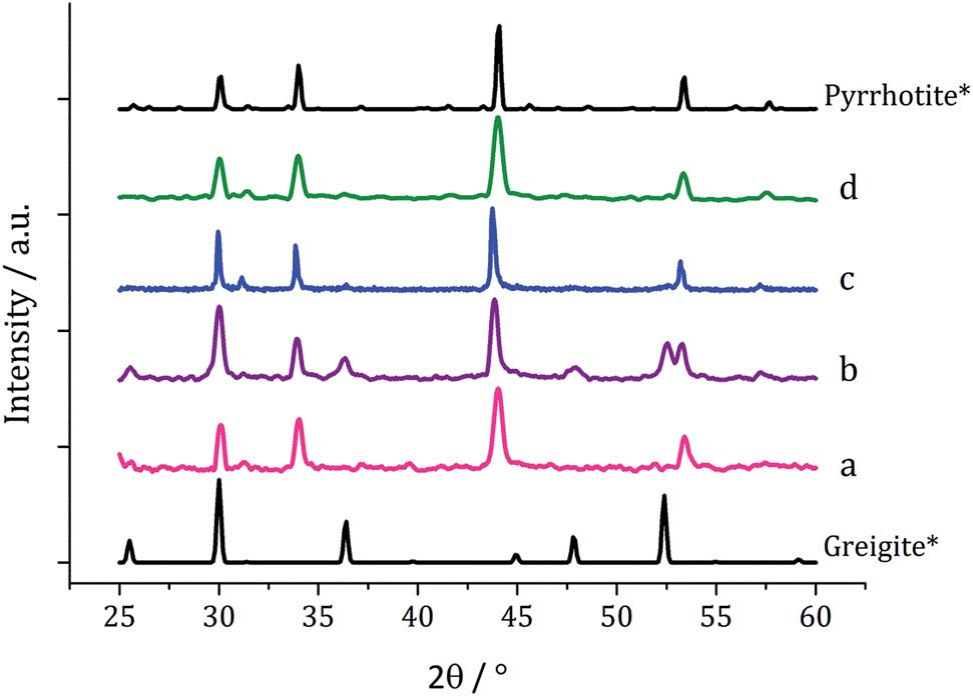
PXRD patterns for nanoparticles obtained from (a) 1a, (b) 1b, (c) 1c and (d) 1d, with reference patterns for bulk greigite (ICDD card no. 16-0713) and pyrrhotite 4M (ICDD card no. 29-0723).

Decompositions were carried out in oleylamine^42^ at 230 °C using the ‘heat-up’ method. Pyrrhotite has been suggested to be thermodynamically more stable than greigite under the conditions employed in this study, and it is also more readily formed upon decomposing certain SSPs.^53^ We initially probed how varying alkyl substituents in 1a–d affects the product. Each gave a dark brown solution upon dissolution in oleylamine, but unexpectedly at 75 °C all turned pale yellow and clear, then at 80 °C they quickly went black. And after 1 h nanoparticles were isolated as black powders. Powder X-ray diffraction (PXRD) analysis revealed that pure pyrrhotite (Fe_7_S_8_, ICDD card no. 029-0723) was formed, except with 1b where a small amount of greigite (ICDD card no. 016-0713) was also observed (Fig. 4). O'Brien has previously found that decomposition of [Fe(S_2_CNRR′)_3_] formed predominantly greigite, but showed peaks for pyrrhotite at higher temperatures (230 and 300 °C).^53^ The lower SSP concentrations used in this work could be a factor as to why the thermally more stable pyrrhotite was predominantly formed.

**Fig. 5:**
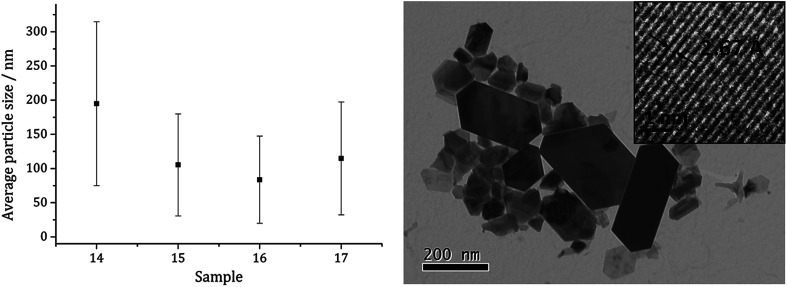
Average particle size (one SD above and below) of samples prepared from 1a–d (left) and TEM image of sample prepared from 1c with HRTEM inset (right).

Average particle size (Fig. 5) decreased as the size of the dithiocarbamate substituents increased; 1c (av. 84 nm) containing the large ^i^Bu substituents. This might suggests that precursors with shorter alkyl chains take longer to decompose, therefore forming larger nanoparticles (less nucleation sites and more growth) and this is supported by the work of O'Brien who found that [Fe(S_2_CNR_2_)_3_] complexes with shorter alkyl chains required higher temperatures to decompose in oleylamine.^53^ High Resolution Transmission Electron Microscopy (HRTEM) of the particles produced from 1c shows spacings of 2.67 Å, consistent with the [004] lattice plane of pyrrhotite-4M (2.64 Å, ICDD card no. 29-0723).

**Fig. 6:**
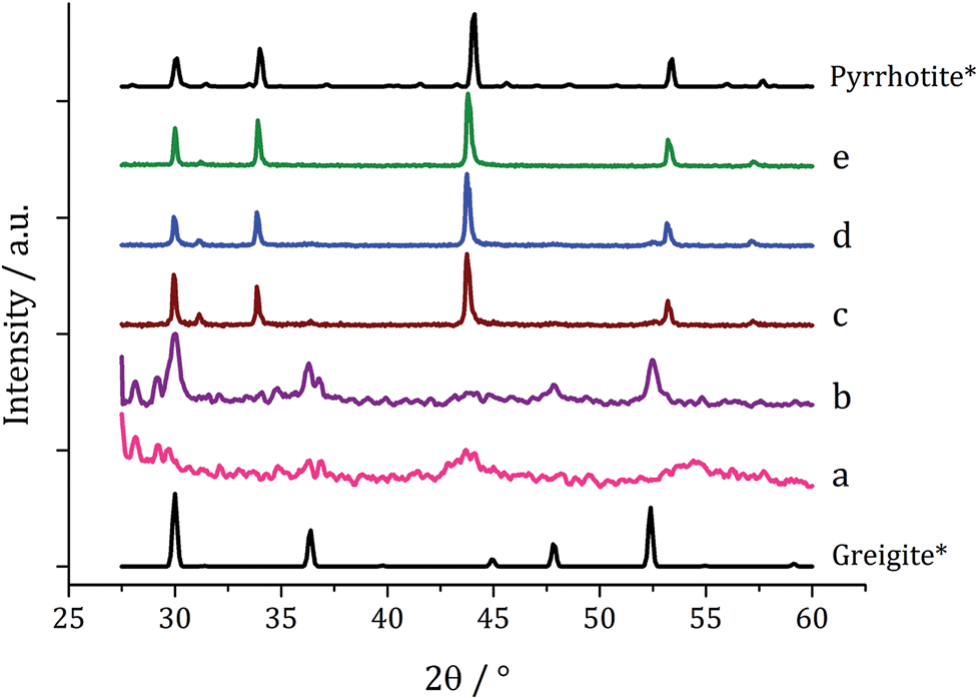
PXRD patterns for samples prepared from 1c at (a) 150 °C, (b) 180 °C, (c) 230 °C, (d) 260 °C and (e) 280 °C, with reference patterns for bulk greigite (ICDD card no. 16-0713) and pyrrhotite 4M (ICDD card no. 29-0723).

We next probed how decomposition of [Fe(S_2_CN^i^Bu_2_)_3_] (1c) was affected by temperature. Solutions (5 mM) in oleylamine (20 mL) were heated for 1 h at 150, 180, 260 and 280 °C respectively and compared *via* PXRD (Fig. 6) to the sample at 230 °C. Those prepared below 230 °C were mostly amorphous, except for some small broad peaks for greigite and pyrrhotite. This is in accordance with Gao^41^ and O'Brien^53^ who both obtained amorphous materials at lower temperatures, with greigite forming at intermediate temperatures, while pyrrhotite was favoured at higher temperatures. Samples prepared at 150 and 180 °C were unstable in air and oxidised to orange-brown powders after 2–3 days suggesting incomplete decomposition of the SSP, whereas all the other samples remained as black powders several months post synthesis.

**Fig. 7:**
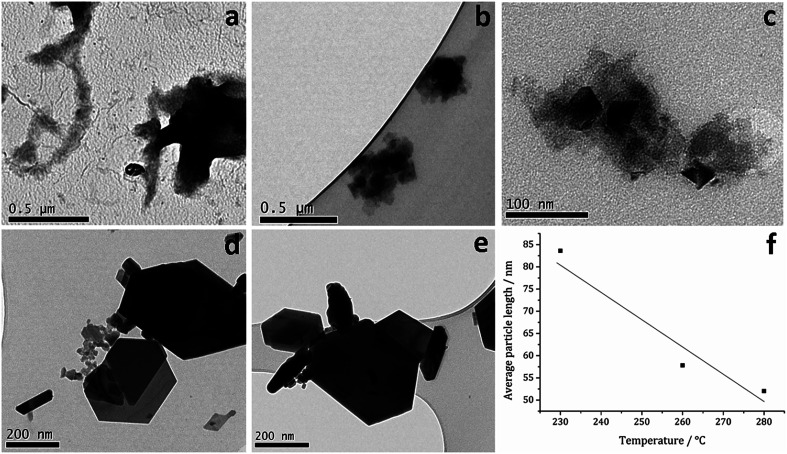
TEM images of samples prepared from 1c at (a) 150, (b) 180, (c) 230, (d) 260 and (e) 280 °C, (f) graph of average particle length against temperature of decomposition.

TEM (Fig. 7) clearly shows the progression from amorphous materials at low temperatures to crystalline material at higher temperature, while nanoparticle shape does not change significantly, being consistent with previous findings.^41,53^ Average particle size decreases as temperature is increased (Fig. 7f) and this could be an effect of the decomposition rate.

**Fig. 8:**
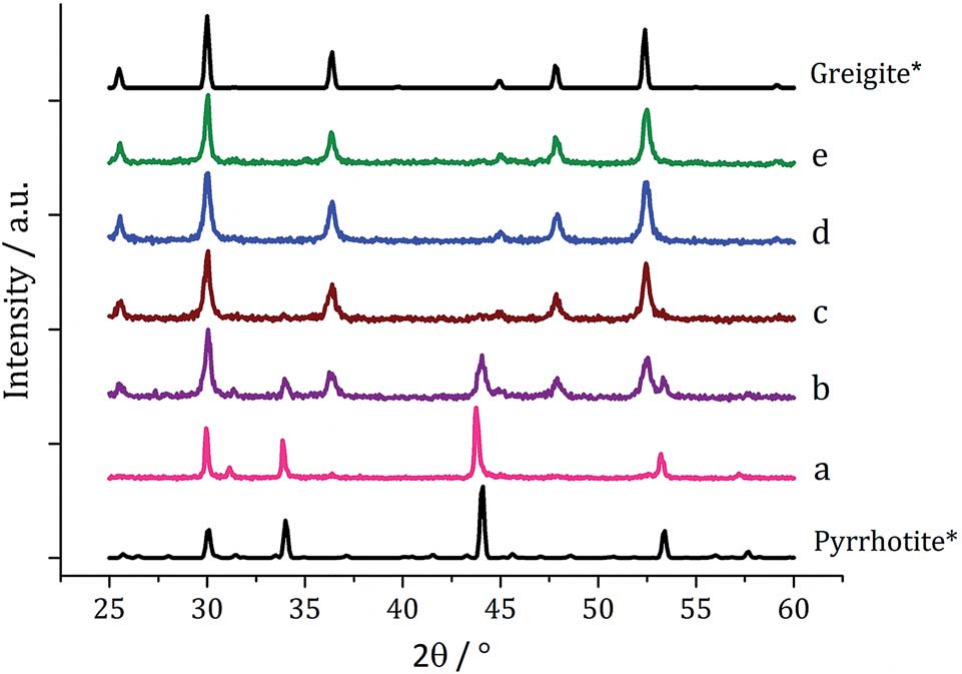
PXRD patterns for samples prepared from 1c at (a) 5, (b) 10, (c) 20, (d) 40 and (e) 50 mM concentration, with reference patterns for bulk pyrrhotite 4M (ICDD card no. 29-0723) and greigite (ICDD card no. 16-0713).

**Fig. 9:**
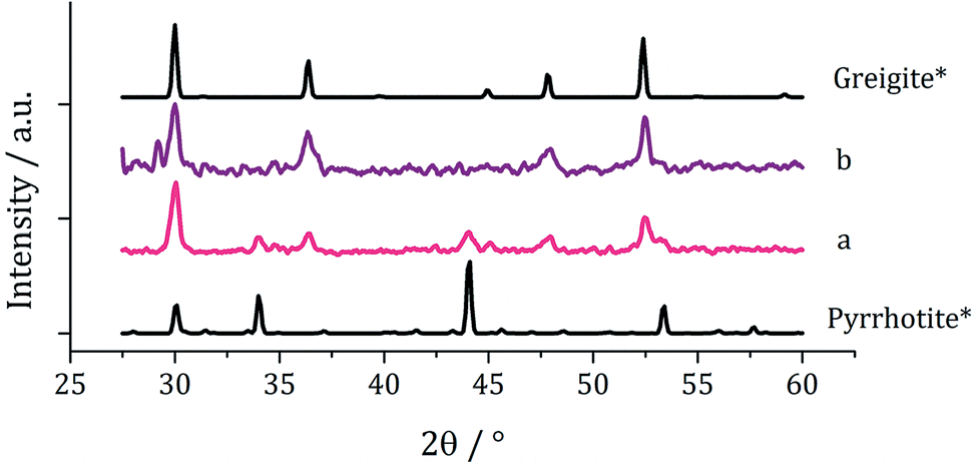
PXRD patterns for samples produced from (a) 2a and (b) 2a with 1a, with reference patterns for bulk pyrrhotite 4M (ICDD card no. 29-0723) and greigite (ICDD card no. 16-0713).

Since at lower temperatures some greigite was formed, in order to access pure greigite in a crystalline form, further decomposition studies were carried out at 230 °C. The concentration of 1c was varied (10–50 mM, in 20 mL oleylamine for 1 h at 230 °C) and the resulting nanomaterials compared with the sample prepared using 5 mM. PXRD analysis shows a progression from pyrrhotite to greigite with increasing concentration (Fig. 8). Formation of pure greigite was possible at 40–50 mM precursor concentrations, consistent with the work of Gao and O'Brien.^41,53^ A HRTEM image of the 40 mM sample (Fig. 9 right), shows *d*-spacings of 2.55 and 2.97 Å, consistent with the [400] and [311] lattice planes of greigite (2.47 and 2.98 Å respectively, ICDD card no. 16-0713). O'Brien has previously studied decomposition of [Fe{SON(CN^i^Pr_2_)_2_}_3_] at varying concentrations.^65^ At 5–10 mM pyrrhotite was formed, but increasing to 20 mM produced an amorphous material, suggesting that concentration plays a role in the crystallinity of the resultant material. The trend observed in the current study, suggests concentration variations may allow access to metastable phases such that at higher concentration the metastable phase greigite is formed.

### (iv) Decomposition of Fe(ii) dithiocarbamate SSPs and Fe(iii)–Fe(ii) mixtures

The work described above shows that it is possible to produce greigite (which contains both Fe(ii) and Fe(iii) centres) from a single Fe(iii) SSP but only within a small range of SSP concentration and decomposition temperature. We thus sought to use Fe(ii) SSPs to potentially widen the range of conditions under which greigite nanomaterials could be produced. We first explored the decomposition of *cis*-[Fe(S_2_CNMe_2_)_2_(CO)_2_] (2a) in oleylamine at 230 °C for 1 h. The complex is only sparingly soluble at room temperature but even upon warming to 35 °C it had fully dissolved to give a dark red-brown solution. The resulting nanoparticles were isolated after cooling the mixture by addition of excess methanol and separation by centrifugation. For comparison a similar decomposition of a 1 : 1 mixture of 2a (2.5 mM) and [Fe(S_2_CNMe_2_)_3_] (1a, 2.5 mM) was carried out, giving a black powder. In both cases, PXRD analysis (Fig. 9) revealed a mixture of greigite and pyrrhotite, although disappointingly the low quality of the patterns indicate that little crystalline material was produced.

**Fig. 10:**
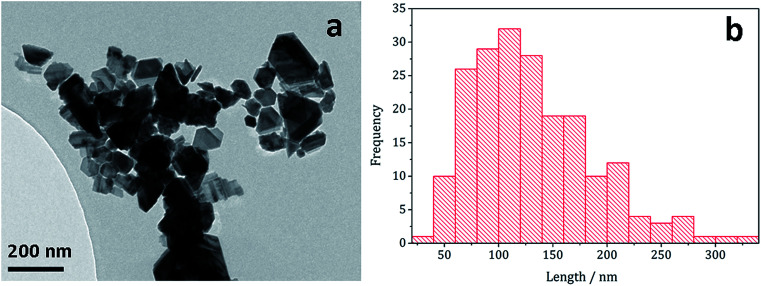
(a) TEM image and (b) histogram of particle length for pyrrhotite particles obtained from decomposition of 1a/2a mixture.

Decomposition of a 1a/2a mixture did not produce greigite. This is surprising as even at 240 °C Gao obtained a mixture of greigite and pyrrhotite from [Fe(S_2_CNEt_2_)_3_] (1b)^41^ and O'Brien also generated a mixture greigite–pyrrhotite at 230 °C.^53^ TEM (Fig. 10) shows hexagonal nanocrystals, similar in appearance to the pyrrhotite synthesised by Gao,^41^ Xu–Wang^42^ and O'Brien,^53^ with a particle diameter range of 20–320 nm, being lower than that obtained from 2a alone.

### (v) Decomposition of [Fe(S_2_CNR_2_)_3_] with added thiuram disulfide

**Scheme 2:**
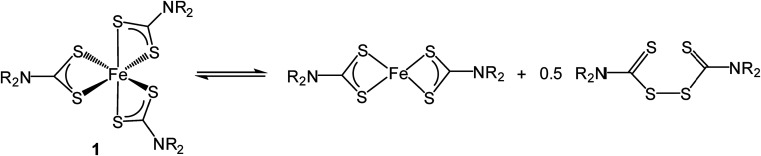
Thermally-induced reduction of [Fe(S_2_CNR_2_)_3_] (1) to give [Fe(S_2_CNR_2_)_2_] and thiuram disulfide.

As briefly communicated^66^ we followed structural changes upon heating [Fe(S_2_CN^i^Bu_2_)_3_] (1c) in oleylamine by *in situ* X-ray absorption spectroscopy (XAS) (discussed in detail later). Pertinent here is the observation that at 60 °C reduction of Fe(iii) to Fe(ii) occurs *via* an intramolecular electron-transfer, associated with concurrent oxidation of dithiocarbamate to thiuram disulfide (Scheme 2). Thus Fe(iii) SSPs actually convert to formation of Fe(ii) species in the decomposition media. Since thiuram disulfides are oxidising agents, adding to low valent metal centres as two dithiocarbamate ligands *via* an oxidative–addition process,^67–72^ we considered that an equilibrium was operating. Hu and Zhang previously considered the role of thiuram disulfides in the solvothermal synthesis of CdS^73^ and we have also recently shown that addition of tetra-iso-butylthiuram disulfide (3) to the oleylamine solutions of [Ni(S_2_CN^i^Bu_2_)_2_] can have a significant effect on the nanomaterials generated.^74^

**Fig. 11:**
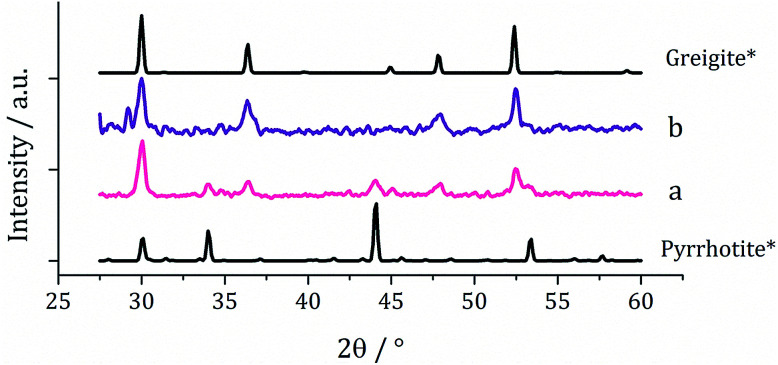
PXRD patterns for samples prepared from 1c (5 mM) with (a) one equivalent and (b) two equivalents of 3, with reference patterns for bulk pyrrhotite 4M (ICDD card no. 29-0723) and greigite (ICDD card no. 16-0713).

One equivalent of 3 was added to 1c (5 mM) and decomposed in oleylamine at 230 °C for 1 h. The materials produced gave off a sulfurous smell, indicating the presence of excess sulfur. The resulting black powder was analysed by PXRD and found to be a mixture of pyrrhotite and greigite (Fig. 11a). Notably, addition of 3 has promoted the stabilisation of the greigite phase. In an attempt to form pure greigite, the decomposition was repeated with two equivalents of 3, and analysis of the resulting particles showed that indeed that pure greigite was formed (Fig. 11b).

**Fig. 12:**
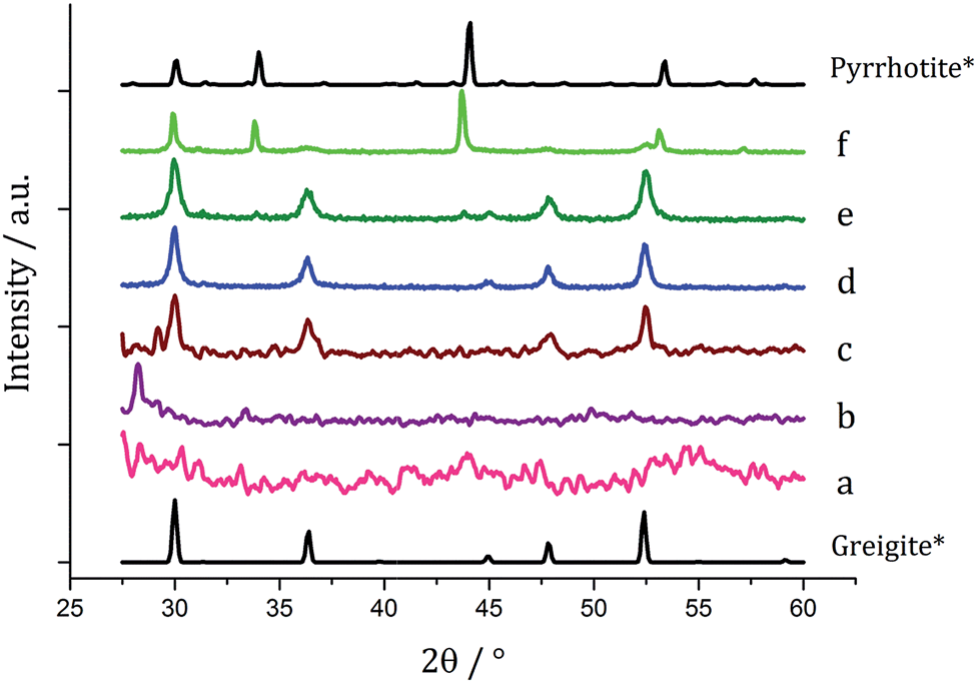
PXRD patterns for samples prepared from 1c (5 mM) and 3 (10 mM) at (a) 150, (b) 180, (c) 230, (d) 260, (e) 280 and (f) 300 °C, with reference patterns for bulk greigite (ICDD card no. 16-0713) and pyrrhotite 4M (ICDD card no. 29-0723).

Based on this result, 1c (5 mM) and 3 (10 mM) were decomposed in oleylamine for 1 h at different temperatures (Fig. 12). At lower temperatures only amorphous materials resulted, but at intermediate temperatures greigite was produced, and with high purity at 260 °C. Above 260 °C, pyrrhotite becomes prevalent, consistent with this being the thermodynamic product. In comparison to decomposition of the SSPs alone, pure crystalline materials can be accessed due to the greater stability of greigite in the presence of 3.

**Fig. 13:**
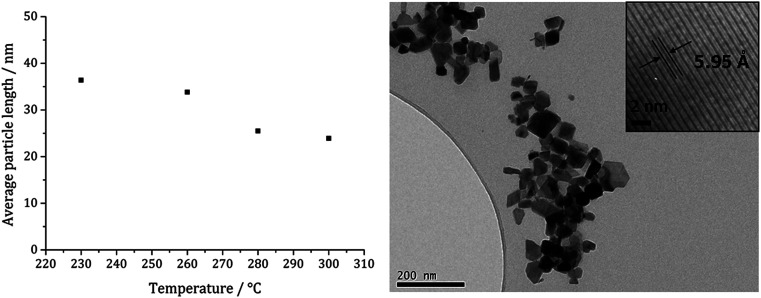
Graph showing the average particle length against temperature of decomposition for precursors 1c (5 mM) and 3 (10 mM) (left). TEM image of sample prepared at 260 °C with HRTEM inset (right).

Fig. 13a shows a graph of the average particle size for samples prepared at 150–300 °C, from which it can be seen that the size decreases slightly with increasing temperature, consistent with the trend seen in the samples prepared without added thiuram disulfide. A TEM image (Fig. 13b) of the material produced at 260 °C shows that while particle morphology is similar to the greigite nanoparticles prepared in the absence of 3, the average particle size of the former is smaller (34 nm as compared to 55 nm respectively). HRTEM of the 260 °C sample (Fig. 13b inset) shows spacings of 5.95 Å, consistent with the [111] lattice plane of greigite, and this temperature was chosen to develop concentration studies as it is the lowest temperature where pure crystalline material is produced.

**Fig. 14:**
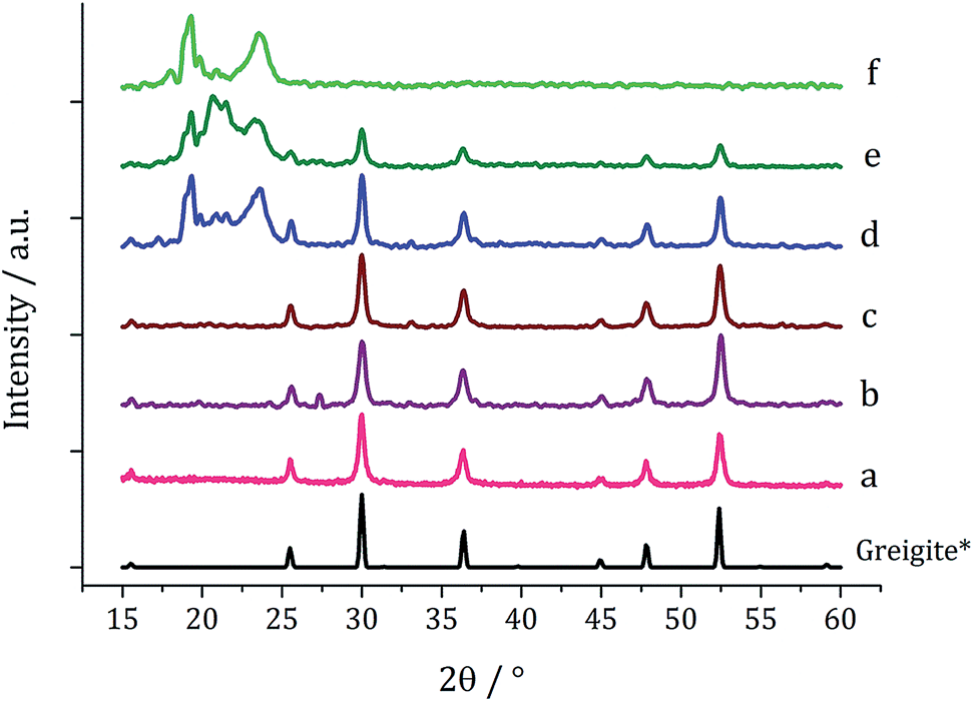
PXRD patterns for samples prepared from 1c and 3 at concentrations of (a) 5 and 10 mM, (b) 10 and 20 mM, (c) 20 and 40 mM, (d) 40 and 80 mM, and (e) 50 mM and 0.5 M, (f) 6 decomposed alone at 80 mM, with reference pattern for bulk greigite (ICDD card no. 16-0713).

When 1c and 3 (in a 1 : 2 ratio) were decomposed at 260 °C (10 : 20, 20 : 40, 40 : 80 and 50 : 100 mM) the black powders formed in all cases were greigite (see PXRD analysis in Fig. 14), although at the higher concentrations there were also anomalous low angle peaks believed to be due to excess sulfur. In order to confirm this, 3 alone was decomposed (80 mM) and the PXRD pattern of the resulting brown powder was a good match to the anomalous peaks seen previously.

**Fig. 15:**
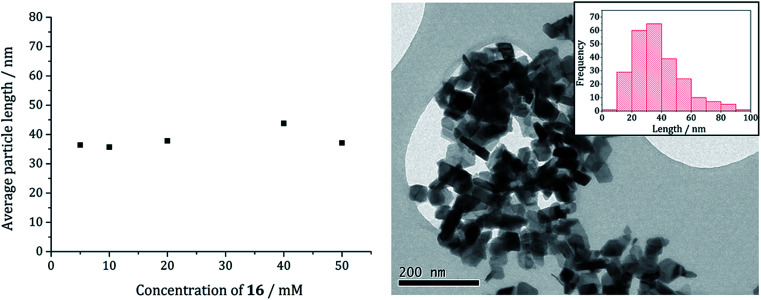
Average particle length of samples prepared from 1c and 3 at various concentrations (in a 1 : 2 ratio) (left). TEM image of greigite prepared from 2d (20 mM) and 1a (40 mM) with particle length histogram inset (right).

The average particle size does not vary significantly with increasing concentration (Fig. 15) suggesting that 3 may be acting also as a capping/stabilising agent, halting particle growth at *ca.* 40 nm. An implication is that greigite can be consistently synthesised at reasonably high precursor concentrations, allowing doping of other metals into the greigite structure, potentially important for future studies.

### (vi)*In situ* XAS studies and plausible decomposition mechanism

In recent work we studied the decomposition of [Ni(S_2_CN^i^Bu_2_)_2_] in hexylamine (HexNH_2_) by *in situ* XAS.^75^ Key findings were; (i) the amine coordinated to the metal centre at low temperatures to afford octahedral complexes [Ni(NH_2_Hex)_2_(S_2_CN^i^Bu_2_)_2_]; (ii) as the temperature was raised the primary amine displaced the ^i^Bu_2_NH *via* amide-exchange resulting in formation of [Ni(S_2_CNHHex)_2_]; (iii) [Ni(S_2_CNHHex)_2_] decomposes at low temperatures *via* deprotonation (accelerated by base) with extrusion of HexNCS. We were also able to support these experimental observations with DFT studies allowing a good overall view of the likely decomposition pathway(s) to be developed. Due to the high-low spin crossover nature of [Fe(S_2_CNR_2_)_3_], related theoretical studies would be complicated and thus we do not consider them here, but note an expectation that high spin complexes would be far more labile than low-spin isomers.

**Fig. 16:**
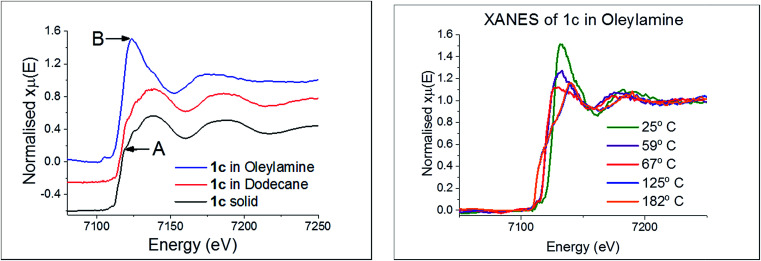
(a) XANES spectra of 1c in various forms, (b) in oleylamine at various temperatures.

**Scheme 3:**
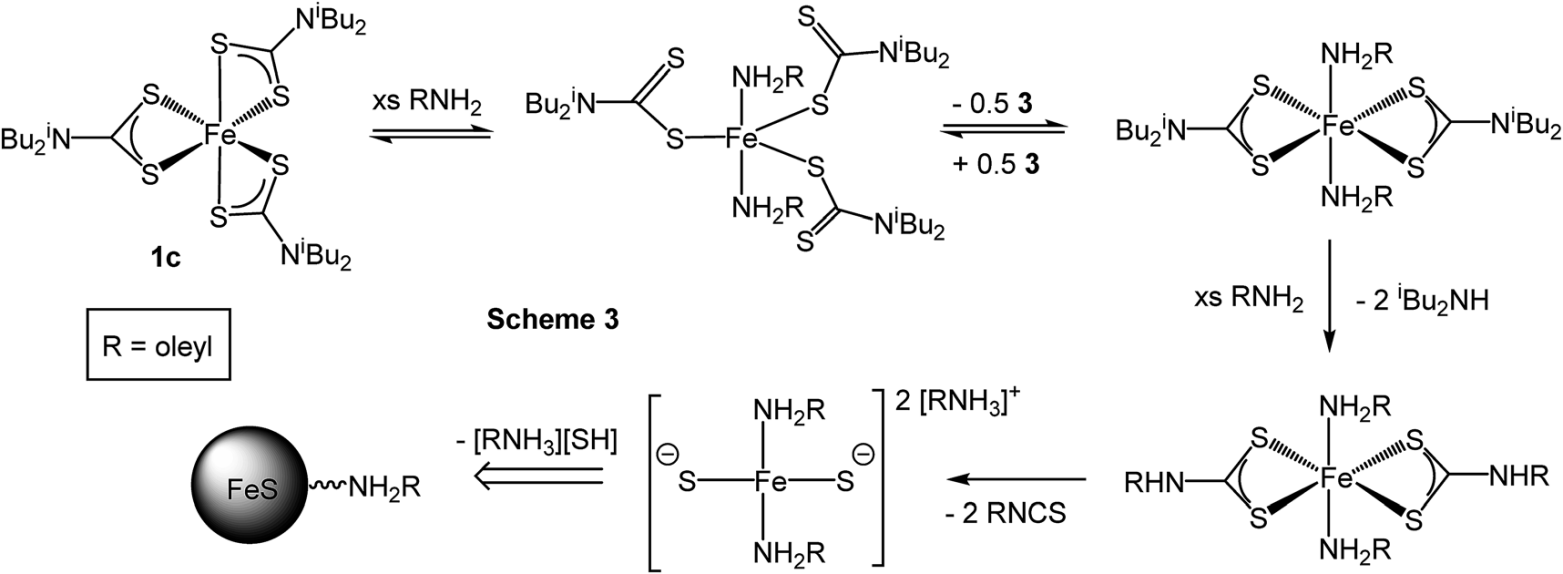
Proposed route for the conversion of [Fe(S_2_CN^i^Bu_2_)_3_] (1c) into iron sulfide nanomaterials upon heating in oleylamine (RNH_2_).

We first considered the molecular structure and thermal stability of [Fe(S_2_CN^i^Bu_2_)_3_] (1c) in the solid-state and in the non-coordinating dodecane. XANES spectra show (Fig. 16a) that the molecular structure is essentially identical under both conditions confirming that the bidentate nature of the dithiocarbamate is maintained upon dissolution in dodecane. Thus EXAFS fitting for 1c in the solid-state correspond well with the single crystal X-ray diffraction data,^76^ showing six Fe–S distances of 2.30 Å. In contrast, dissolution in oleylamine afforded a quite different XANES spectrum; white line intensity at *ca.* 7124 eV (labelled B) being far more prominent that in the solid-state or dodecane, while the shoulder at *ca.* 7119 eV (labelled A) in the latter two spectra associated with the p-orbital contribution of sulfur to the 4s orbital of iron is much reduced in oleylamine. This provides strong evidence that the local octahedral tris(dithiocarbamate) structure is not maintained in oleylamine. Modelling of 1c in oleylamine strongly suggests that, upon coordination of the amine, the dithiocarbamate ligands become monodentate, and best fit supports a model with a five-coordinate trigonal bipyramidal iron centre ligated by two amines in the axial sites (Fe–N 1.89 Å) with three monodentate dithiocarbamate ligands (Fe–S 2.21 and 3.08 Å) occupying the basal positions.^66^ Thus upon dissolution in oleylamine 1c is actually best considered as [Fe(κ_1_-S_2_CN^i^Bu_2_)_3_(RNH_2_)_2_] (R = oleyl) (Scheme 3). Amine binding might also explain the significantly enhanced solubility of [Fe(S_2_CNMe_2_)_3_] in oleylamine above 35 °C.

*In situ* XAS studies were carried out on the decomposition of 1c in oleylamine. For these experiments we attempted to replicate the laboratory conditions as best we could and we used the same concentrations of all reagents. Nevertheless, the cell design does not allow for a mixing mechanism, and thus the potential for larger nanomaterials to be deposited at the bottom of the cell cannot be discounted. Further the cell is closed and thus venting of generated gases cannot occur, while heat loss across the cell in this geometry is inevitable since there is a small cell body at the centre which faces ambient conditions. To counter this we performed temperature calibrations prior to experiments using a secondary thermocouple accessed through a small hole in the reaction chamber, thus we believe that we had a good estimate of temperature in the reaction chamber at all times.

Little change occurred in the XANES spectra up to *ca.* 70 °C, suggesting that the amine-adduct remains intact. Above 70 °C an edge shift was apparent (Fig. 16b) being indicative of reduction of Fe(iii) to Fe(ii). EXAFS analysis supports an octahedral Fe(ii) centre ligated by two chelating dithiocarbamate ligands and two amines; [Fe(S_2_CN^i^Bu_2_)_2_(RNH_2_)_2_] (Scheme 3). This fits well with the decomposition studies, where between *ca.* 75 °C the intense brown solution become pale yellow very suddenly; we associate this change with reduction of the iron centre. Previous work on related xanthate complexes, [Fe(S_2_COR)_3_], showed a similar Fe(iii) to Fe(ii) reduction upon addition of pyridine, the products being [Fe(S_2_COR)_2_(py)_2_] and (ROCS_2_)_2_.^77^ Reduction of Fe(iii) to Fe(ii) involves intramolecular electron-transfer with concomitant oxidation of dithiocarbamate to thiuram disulfide 3. While we have not carried out XAS studies on iron(ii) dicarbonyl complexes, 2, a similar scheme may be invoked; that is thermal loss of both carbonyls generating square-planar [Fe(S_2_CNR_2_)_2_] *in situ* which rapidly coordinates amine to afford the same intermediate. Thus Fe(ii) and Fe(iii) SSPs are likely generating the same molecular precursors in the amine solution.

Between 67 and 125 °C XAS data was lost due to the severe inhomogeneity of the reaction mixture; showing that it is between these temperatures that nanoparticle formation occurs. Consequently we cannot confidently comment directly on whether amide-exchange seen previously at a Ni(ii) centre,^75^ whereby [Ni(S_2_CN^i^Bu_2_)_2_] is converted into [Ni(S_2_CNHR)_2_)] occurs at the Fe(ii) centre. However, we would expect that changing from a square-planar Ni(ii) to Fe(ii) centre would not significantly alter the reactivity of the bound ligands (we realise that a d8 square planar geometry for Ni(ii) is probable, while for d6 Fe(ii) a low spin octahedral arrangement is attractive), and thus we propose a related exchange occurs rapidly after the electron-transfer to afford [Fe(RNH_2_)_2_(S_2_CNHR)_2_] (Scheme 3). The rapid nature of the nanoparticle formation above 60 °C strongly suggests that amide-exchange is fast at the Fe(ii) centre.

We have previously shown that nickel complexes with primary amine backbones decompose at much lower temperatures than those with secondary amines due to the base-mediated deprotonation of the backbone proton(s) and subsequent extrusion of organic isothiocyanate.^75^ Thus we would expect [Fe(RNH_2_)_2_(S_2_CNHR)_2_] to rapidly extrude oleylisocyanate (RNCS) (two equivalents shown) to afford a small molecular precursor that can nucleate to give the observed iron sulfide nanomaterials (Scheme 3).

This then leads us to return to consider how addition of thiuram disulfide 3 affects the decomposition mechanism; allowing greigite to be formed under conditions where in its absence pyrrhotite formation is favoured. As discussed earlier, the most obvious point of entry of thiuram disulfide is at the Fe(iii)–Fe(ii) redox transformation, as clearly addition should move the equilibrium towards Fe(iii). Especially if reduction is rate-limiting then addition of 3 should have a significant effect. The second place it can be potentially important is later in the transformation as a sulfur source. Thus purported “FeS(OA)_*x*_” fragments are anticipated to be highly reactive and may be able to abstract sulfur from 3 to give the corresponding thiuram monosulfide. This would also result in oxidation of the metal centre and thus account for both the increased sulfur content and overall metal oxidation state seen in greigite (Fe_3_S_4_) *versus* pyrrhotite (Fe_1–*x*_S).

## Summary and conclusions

Solvothermal decomposition of iron(ii) and iron(iii) dithiocarbamate SSPs has been thoroughly and systematically investigated by changing a range of reaction variables in order to probe the changes to materials formed. Iron(ii) dithiocarbamate SSPs were not required to produce iron sulfide nanoparticles containing Fe(ii) ions since they can be generated upon reduction of Fe(iii) species. This process is relatively fast as shown when the decomposition of [Fe(S_2_CNBu^i^_2_)_3_] (1c) was halted immediately upon reaching the decomposition temperature pure greigite resulted. However, the greigite formed is a metastable phase and, if enough energy is supplied, pyrrhotite will forms. Greigite formation can be promoted by reducing the decomposition time, lowering the temperature and increasing the precursor concentration. All these factors decrease the amount of energy transferred to each precursor unit and so prevent the formation of the more thermodynamically favoured phase (pyrrhotite). In addition, precursor choice can have an effect. Thus [Fe(S_2_CNEt_2_)_3_] (1b) showed a greater propensity towards the formation of greigite, which may be due to a different decomposition mechanism being at work, or to the formation of a by-product that stabilises the greigite phase.

Addition of thiuram disulfide (3) to the decomposition system had a significant effect, such that at high concentrations of 3 greigite could be prepared at higher temperatures and lower concentrations of 1c. Reasons for this difference are not clear but could relate to relative ratios of Fe(iii) and Fe(ii) species in the decomposition mixture which affects the overall decomposition mechanism. Decomposition of the Fe(ii) precursor 2a produces pure pyrrhotite (Fe_7_S_8_) nanoparticles with similar morphology to those previously synthesised from other iron(ii) dithiocarbamate precursors. The addition of an Fe(iii) source 1a, did not lead to the formation of greigite as predicted, but rather pure pyrrhotite resulted. Other groups have been able to access greigite by decomposing Fe(S_2_CNR_2_)_3_, although in many cases with pyrrhotite impurities.^8,36,37^

Attempts are ongoing to better understand the molecule to materials mechanism as a pattern develops between the decomposition pathway(s) of a range of transition metal dithiocarbamate complexes in amine solutions. For work on the potential role of iron sulfides in prebiotic chemistry,^25^ the ability to prepare greigite samples of high purity and varying average sizes allows us to probe how the latter affects their activity and also the effects of doping other metal ions (especially nickel) into the greigite structure on activity.

## Experimental section

### General procedures

All manipulations were performed under a dry, oxygen-free dinitrogen atmosphere using standard Schlenk techniques or in a MBRAUN Unilab glovebox. All solvents used were stored in alumina columns and dried with anhydrous engineering equipment, such that the water concentration was 5–10 ppm. All other reagents were procured commercially from Aldrich and used without further purification. Microanalytical data was obtained at UCL. Thiuram disulfide 3 was prepared as previously reported.^74^

### Physical measurements

^1^H and ^13^C{^1^H} NMR spectra were obtained on either a Bruker Avance III 400 or Avance 600 spectrometers. All spectra were recorded using CDCl_3_ which was dried and degassed over molecular sieves prior to use; ^1^H and ^13^C{^1^H} chemical shifts are reported relative to SiMe_4_. Mass spectra were obtained using either Micromass 70-SE spectrometer using Electron Ionisation (EI) or a Thermo Finnigan MAT900xp spectrometer using Fast Atom Bombardment (FAB) ionisation. Elemental analysis was carried using Elemental Analyser (CE-440) (Exeter Analytical Inc). Thermogravimetric analysis (TGA) was performed using a Netzsch STA 449C TGA system. Data was recorded from 25 to 600 °C with a constant heating rate of 10 °C per minute. XRD were measured on a Bruker AXS D4 diffractometer using CuKα_1_ radiation. The diffraction patterns obtained were compared to database standards. For TEM characterisation a 4 μL droplet of nanoparticle suspension (chloroform) was placed on a holey carbon-coated copper TEM grid and allowed to evaporate in air under ambient laboratory conditions for several minutes. TEM images were obtained using a JEOL-1010 microscope at 100 kV equipped with a Gatan digital camera. HRTEM measurements were collected using a Jeol 2100 (high resolution) TEM with a LaB_6_ source operating at an acceleration voltage of 200 kV. Micrographs were taken on a Gatan Orius charge-coupled device (CCD).

### Synthesis and characterisation of [Fe(S_2_CNR_2_)_3_] (1)

#### [Fe(S_2_CNMe_2_)_3_] (1a)^54^

NaS_2_CNMe_2_ (4.30 g, 30 mmol) in water (60 mL) was added dropwise to a solution of FeCl_3_ (1.62 g, 10 mmol) in water (50 mL), whereupon a black precipitate formed. This mixture was vigorously stirred for 2 h, filtered, washed with water (3 × 30 mL) and evaporated to dryness. The resulting black powder was dissolved in 100 mL of CH_2_Cl_2_ and stirred with magnesium sulphate for 30 min, after which it was filtered and the filtrate dried *in vacuo*. Yield 3.29 g, 79%. Anal. calc. for C_9_H_18_N_3_S_6_Fe: C, 25.95; H, 4.36; N, 10.09. Found: C, 25.79; H, 4.37; N, 10.17. MS: *m*/*z* 416 [M^+^], 296 [M^+^ − C_3_H_6_NS_2_]. IR (*ν*_max_ cm^−1^): 1516 (s) [N=C], 972 (s), 1247 (s) [C=S], 1137 (s) [C_2_N].

#### [Fe(S_2_CNEt_2_)_3_] (1b)

Prepared following the same method as with 1a, with the exception that NaS_2_NCEt_2_ (6.76 g, 30 mmol) was used. Yield 4.30 g, 86%. Anal. calc. for C_15_H_30_N_3_S_6_Fe: C, 35.99; H, 6.04; N, 8.39. Found: C, 35.88; H, 6.01; N, 8.40. MS: *m*/*z* 500 [M^+^], 352 [M^+^ − C_5_H_10_NS_2_]. IR (*ν*_max_ cm^−1^): 1485 (s) [N=C], 994 (s), 1270 (s) [C=S], 1133 (s) [C_2_N].

#### [Fe(S_2_CN^i^Bu_2_)_3_] (1c)

^i^Bu_2_NH (5.24 mL, 30 mmol) was added to NaOH (1.20 g, 30 mmol) in water (50 mL). To this mixture CS_2_ (1.80 mL, 30 mmol) was added dropwise over 10 min and the mixture stirred overnight. A solution of FeCl_3_ (1.62 g, 10 mmol) in water (50 mL) was added dropwise over 5 min, whereupon a black precipitate formed. This mixture was vigorously stirred for 2 h, filtered, washed with water (3 × 30 mL) and evaporated to dryness. The resulting black powder was dissolved in 100 mL of CH_2_Cl_2_ and stirred with magnesium sulphate for 30 min, after which the mixture was filtered and the filtrate dried *in vacuo*. Yield 5.55 g, 83%. Anal. calc. for C_27_H_54_N_3_S_6_Fe: C, 48.48; H, 8.14; N, 6.23. Found: C, 48.52; H, 8.26; N, 6.23. MS: *m*/*z* 669 [M^+^], 464 [M^+^ − C_9_H_18_NS_2_]. IR (*ν*_max_ cm^−1^): 1482 (s) [N=C], 992 (s), 1244 (s) [C=S], 1145 (s) [C_2_N].

#### [Fe(S_2_CNMeBu)_3_] (1d)

Prepared following the same method as with 1c, with the exception that MeBuNH (3.55 mL, 30 mmol) was used. Yield 0.39 g, 43%. Anal. calc. for C_9_H_18_N_3_S_6_Fe: C, 39.83; H, 6.69; N, 7.74. Found: C, 39.54; H, 6.83; N, 7.61. MS: *m*/*z* 543 [M^+^], 380 [M^+^ − C_6_H_12_NS_2_]. IR (*ν*_max_ cm^−1^): 1496 (s) [N=C], 936 (s), 1246 (s) [C=S], 1144 (s) [C_2_N].

### Synthesis and characterisation of [Fe(S_2_CNR_2_)_2_(CO)_2_] (2)

#### [Fe(CO)_2_(S_2_CNMe_2_)_2_] (2a)^60^

Fe(CO)_4_I_2_ was synthesised and used *in situ*. A solution of iodine (0.38 g, 1.5 mmol) in Et_2_O (10 mL) was added dropwise to a solution of Fe(CO)_5_ (0.20 mL, 1.5 mmol) also in Et_2_O (10 mL). After stirring for 15 min Fe(CO)_4_I_2_ was detected by IR *ν*(CO) cm^−1^: 2137, 2090, 2072. A CH_2_Cl_2_ solution (20 mL) of [H_2_NMe_2_][S_2_CNMe_2_] (0.50 g, 3.0 mmol) was added dropwise to Fe(CO)_4_I_2_ and the mixture stirred for 18 h. The product was separated from [H_2_NMe_2_]I salt by cannula filtration and layered with heptane (10 mL) to yield copper-coloured crystals. Yield 0.24 g, 45% ^1^H NMR *δ*/ppm (CDCl_3_): 3.21 (s, 6H, C*H*_3_), 3.28 (s, 6H, C*H*_3_). ^13^C{^1^H} NMR *δ*/ppm (CDCl_3_): 38.3, 38.7 (*C*H_3_), 206.9 (*C*S_2_), 212.9 (*C*O). Anal. calc. for C_8_H_12_N_2_S_4_O_2_Fe: C, 27.27; H, 3.43; N, 7.95. Found: C, 27.92; H, 3.65; N, 7.44. MS: *m*/*z* 352 [M^+^], 296 [M^+^ − 2CO]. IR *ν*(CO) cm^−1^: 2023, 1967.

#### [Fe(S_2_CNEt_2_)_2_(CO)_2_] (2b)

To a solution of Fe(CO)_4_I_2_ a CH_2_Cl_2_ (20 mL) solution of [NMe_4_][S_2_CNEt_2_] (1.07 g, 4.8 mmol) was added dropwise and the mixture stirred for 18 h. The solid product was extracted in toluene (10 mL) and layered with heptane (10 mL) to give a copper-coloured oil. A dry powder was obtained by washing with pentane (5 mL). Yield 0.26 g, 42%. ^1^H NMR *δ*/ppm (CDCl_3_): 1.27 (m, 12H, CH_2_C*H*_3_), 3.73 (m, 8H, C*H*_2_CH_3_). ^13^C{^1^H} NMR *δ*/ppm (CDCl_3_): 12.6, 12.6, 12.8 (CH_2_*C*H_3_), 43.4, 43.8, 43.8 (*C*H_2_CH_3_), 205.8 (*C*S_2_), 213.1 (*C*O). Anal. calc. for C_12_H_20_N_2_S_4_O_2_Fe: C, 35.29; H, 4.94; N, 6.86. Found: C, 36.00; H, 5.08; N, 6.76. MS: *m*/*z* 393 [M^+^ − CH_3_] 352 [M^+^ − 2CO]. IR *ν*(CO) cm^−1^: 2022, 1966.

#### [Fe(S_2_CN^i^Bu_2_)_2_(CO)_2_] (2c)

To an Et_2_O solution of Fe(CO)_4_I_2_ was added an Et_2_O (10 mL) solution of [NMe_4_][S_2_CN^i^Bu_2_] (1.00 g, 3 mmol) and the mixture stirred for 18 h. The solid product was extracted in hexane (10 mL) and cooled to −10 °C to obtain copper-coloured crystals. Yield 0.34 g, 43%. ^1^H NMR *δ*/ppm (CDCl_3_): 0.93 (m, 24H, C*H*_3_), 2.17 (m, *J* = 6.9 Hz, 2H, C*H*), 2.23 (m, *J* = 6.9 Hz, 2H, C*H*), 3.52 (m, 8H, C*H*_2_). ^13^C{^1^H} NMR *δ*/ppm (CDCl_3_): 20.2, 20.3, 20.3, 20.4 (*C*H_3_), 27.1, 27.2 (*C*H), 58.9, 56.4 (*C*H_2_), 208.1 (*C*S_2_), 213.1 (*C*O). MS: *m*/*z* 464 [M^+^ − 2CO]. IR *ν*(CO) cm^−1^: 2026, 1974.

#### [Fe(S_2_CNMeBu)_2_(CO)_2_] (2d)

To an Et_2_O solution of Fe(CO)_4_I_2_ a solution of [NMe_4_][S_2_CNMeBu] (0.75 g, 3 mmol) in CH_2_Cl_2_ (20 mL) was added dropwise and the mixture stirred for 15 min. The product was extracted in toluene (10 mL) and layered with heptane (10 mL) but no crystals could be obtained from the oily product. Extraction with pentane (5 mL) yielded a brown powder after two weeks in the freezer. Yield 0.22 g, 34%. ^1^H NMR *δ*/ppm (CDCl_3_): 0.95 (m, 6H, CH_2_(CH_2_)_2_C*H*_3_), 1.50 (m, 8H, CH_2_(C*H*_2_)_2_CH_3_), 3.19 (m, 6H, NCH_3_), 3.60 (m, 6H, C*H*_2_(CH_2_)_2_CH_3_). ^13^C{^1^H} NMR *δ*/ppm (CDCl_3_): 13.9, 13.9, 14.0 ((CH_2_)_3_*C*H_3_), 19.9, 20.0, 20.1 (*C*H_2_), 29.0, 29.1, 29.2 (*C*H_2_), 36.2, 36.6, (*C*H_3_), 50.9, 51.1, 51.4 (*C*H_2_), 206.5 (*C*S_2_), 213.0 (*C*O). Anal. calc. for C_18_H_36_N_2_S_4_Ni: C, 38.53; H, 5.54; N, 6.42. Found: C, 38.84; H, 5.20; N, 6.03. MS: *m*/*z* 381 [M^+^ − 2CO]. IR *ν*(CO) cm^−1^: 2036, 1967.

### Decomposition studies

In a typical synthesis the dithiocarbamate complex (5 mM) was added to oleylamine (20 mL) in a three-neck round bottom flask attached to a water condenser and evacuated and refilled with nitrogen repeatedly for *ca.* 15 minutes. The solution was heated to 230 °C and held there for 1 h. The mixture was allowed to cool to room temperature slowly, whereupon methanol (80 mL) was added with stirring. The mixture was centrifuged and the solution decanted leaving behind the resultant nanoparticles. This procedure was repeated three times and then the material was dried under vacuum. A picture of the apparatus used is given in the ESI.‡

### XAS studies

XAS spectra were acquired at the iron K-edge (7112eV) on the Dutch-Belgian EXAFS beamline, BM26A.^78^ Monochromatic radiation was supplied by a double Si(111) crystal, ion chambers were used to measure incident and transmitted beam intensities (*I*_0_ and *I*_t_), and fluorescence was measured using a 9 element germanium solid state detector. Solid 1c was diluted with polyvinylpyrrolidone, pelletized, and placed in the beam. Solutions of 1c were held within *in situ* liquid cells. The *in situ* liquid cell used was developed at UCL for synchrotron based experiments on liquid samples. Cartridge heaters embedded into the conductive cell body allow temperatures to reach up to 200 °C, subject to pressure buildup of the system. The Kapton sealed reaction chamber holds 400 μL of solution with a fixed path length of 2 mm. Measurements were taken in fluorescence. The *in situ* liquid microtron cell was developed by Sample Environment at the ESRF for liquid experiments up to 260 °C. With quartz cell windows and a 4mm path length, the cell was used for measurements in transmission. XAS data was normalized and background subtracted using Horae Athena software.^79^ Linear combination analyses were also performed using Horae Athena. Detailed EXAFS analyses were performed on Excurve Version 9.273.^80^

## Conflicts of interest

There are no conflicts of interest to declare.

## Supplementary Material

NA-001-C9NA00262F-s001
