# Supplementary material for: Fe(ii) and Fe(iii) dithiocarbamate complexes as single source precursors to nanoscale iron sulfides: a combined synthetic and in situ XAS approach
Source: Nanoscale Adv. 2019 Jun 5;1(8):2965–78. doi: 10.1039/c9na00262f (PMC9419884; doi:10.1039/c9na00262f)

Electronic Supplementary Material (ESI) for Nanoscale Advances.
This journal is © The Royal Society of Chemistry 2019

Fe(II) and Fe(III) dithiocarbamate complexes as single source precursors to nanoscale iron sulfides: A combined synthetic and *in-situ* XAS approach

Anna Roffey,[a,b] Nathan Hollingsworth[b], Husn-Ubayda Islam[b,c], Wim Bras[c,d], Gopinathan Sankar[b], Nora De Leeuw[e], and Graeme Hogarth[a*]

[a] *Department of Chemistry, King's College London, Britannia House, 7 Trinity Street, London SE1 1DB, UK*

[b] *Department of Chemistry, University College London, 20 Gordon Street, London WC1H OAJ, UK*

[c] *Netherlands Organisation for Scientific Research DUBBLE@ESRF, 38043 Grenoble, France*

[d] *Chemistry Division, Oak Ridge National Laboratory, Oak Ridge, Tennessee 37831, US*

[e] *School of Chemistry, Cardiff University, Main Building, Park Place, Cardiff, CF10 3AT, UK*

Synthesis of dithiocarbamate salts

[H_2NMe_2][S_2CNMe_2] Me_2NH (15 mmol, 7.50 mL of a 2M solution in THF) was dissolved in further THF (20 mL) CS_2 (0.45 mL, 7.5 mmol) was added slowly dropwise over five mins whereupon a white precipitate began to form. The mixture was stirred for two h, filtered using a Büchner funnel and washed with THF (3 x 10 mL). Yield 1.1200 g, 90 %. **^{1}H NMR δ/ppm ($CDCl_3$):** 2.78 (s, 6H, CH_3), 3.58 (s, 6H, CH_3), 8.42 (broad s, 2H, [$(CH_3)_2NH_2$]). **^{13}C{^{1}H} NMR δ/ppm ($CDCl_3$):** 35.4 (CH_3), 44.8 (CH_3), 210.2 (CS_2). **Anal. Calc. for $C_5H_{14}N_2S_2$:** C, 36.11; H, 8.49; N, 16.84. Found: C, 35.56; H, 8.38; N, 16.57. **MS(EI):** *m/z* 121 [$S_2CN(CH_3)_2 + H^+$].

[NMe_4][S_2CNEt_2] To an ice-cooled solution of Me_4NOH (10 mmol, 3.65 g of a 25% wt solution in methanol) and Et_2NH (10 mmol) in methanol (50 mL), CS_2 (10 mmol, 0.60 mL) was added dropwise over five mins. The solution was allowed to come to room temperature and stirred overnight. Methanol was removed *in vacuo* resulting in a pale yellow crystalline powder. Yield 2.2075 g, 99 %. **^{1}H NMR δ/ppm ($CDCl_3$):** 1.23 (t, 6H, *J*

= 7.0 Hz, CH_2CH_3), 4.14 (q, 4H, *J* = 7.0 Hz, CH_2CH_3). **$^{13}C\{^1H\}$ NMR δ/ppm ($CDCl_3$):** 12.7 (CH_2CH_3), 47.5 (CH_2CH_3), 56.2 ($N(CH_3)_4$) 210.6 (CS_2). **Anal. Calc. for $C_9H_{22}N_2S_2$:** C, 48.60; H, 9.97; N, 12.06. Found: C, 48.41; H, 9.54; N, 11.73. **MS(EI):** *m/z* 148 [$S_2CN(CH_2CH_3)_2$].

[NMe_4][$S_2CNMeBu$] To an ice-cooled solution of Me_4NOH (10 mmol, 3.65 g of a 25% wt solution in methanol) and MeBuNH (10 mmol) in methanol (50 mL), CS_2 (10 mmol, 0.60 mL) was added dropwise over five mins. The solution was allowed to come to room temperature and stirred overnight. Methanol was removed *in vacuo* resulting in a pale yellow crystalline powder. Yield 1.1578 g, 98 %. **^1H NMR δ/ppm ($CDCl_3$):** 0.91 (t, 3H, *J* = 7.4 Hz, $S_2CN(CH_3)(CH_2)_3CH_3$), 1.31 (m, 2H, CH_2), 1.65 (m, 2H, CH_2), 3.51 (s, 3H, $S_2CN(CH_3)(CH_2)_3CH_3$), 3.55 (s, 12H, $N(CH_3)_4$), 4.14 (m, 2H, CH_2). **$^{13}C\{^1H\}$ NMR δ/ppm ($CDCl_3$):** 14.19 ($S_2CN(CH_3)(CH_2)_3CH_3$), 20.32 (CH_2), 29.44 (CH_2), 42.41 (CH_2), 59.00 ($S_2CN(CH_3)(CH_2)_3CH_3$), 56.17 ($N(CH_3)_4$) 211.77 (CS_2). **Anal. Calc. for $C_{10}H_{24}N_2S_2$:** C, 50.80; H, 10.23; N, 11.85. Found: C, 49.47; H, 10.13; N, 11.41. **MS(EI):** *m/z* 162 [$S_2CN(CH_3)(CH_2)_3CH_3$].

Apparatus used for decomposition studies

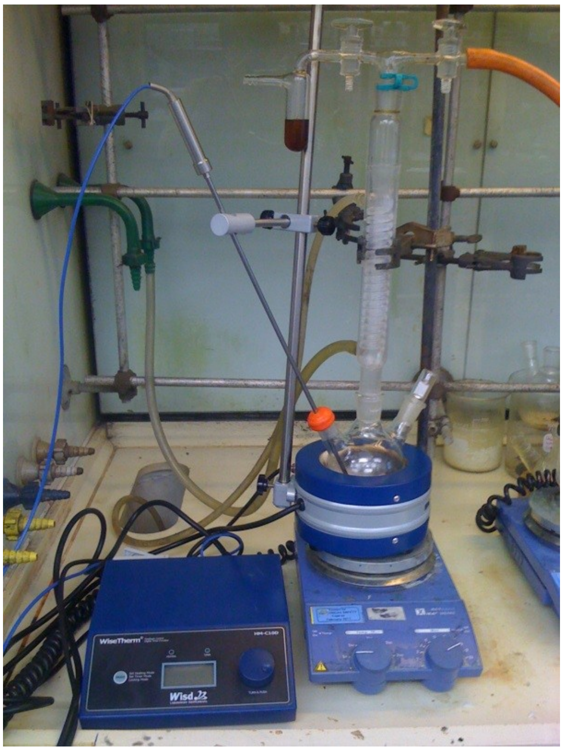

Supplement: NA-001-C9NA00262F-s001 [file NA-001-C9NA00262F-s001.pdf]
